# Epidemiology and Molecular Characterization of Human Respiratory Syncytial Virus in Senegal after Four Consecutive Years of Surveillance, 2012–2015

**DOI:** 10.1371/journal.pone.0157163

**Published:** 2016-06-17

**Authors:** Amary Fall, Ndongo Dia, El Hadj Abdel Kader Cisse, Davy E. Kiori, Fatoumata Diene Sarr, Sara Sy, Debora Goudiaby, Vincent Richard, Mbayame Ndiaye Niang

**Affiliations:** 1 Institut Pasteur de Dakar, Unité de Virologie Médicale, Dakar, Sénégal; 2 Institut Pasteur de Dakar, Unité d’Epidémiologie des maladies infectieuses, Dakar, Sénégal; Kliniken der Stadt Köln gGmbH, GERMANY

## Abstract

**Background:**

The burden of respiratory syncytial virus (RSV) infection remains poorly defined in Africa. To address this, we carried out a descriptive and retrospective pilot study, with a focus on the epidemiology of RSV in Senegal after 4 years of surveillance.

**Methodology and Results:**

From January 2012 to October 2015 swabs were collected from consenting ILI outpatients. Viral detection was performed using RV16 kit enabling direct subtyping of RSV-A and B. For the molecular characterization of HRSV, the second hypervariable region of the Glycoprotein (G) gene was targeted for sequencing. We enrolled 5338 patients with 2803 children younger than five years of age (52.5%). 610 (11.4%) were positive for RSV infection: 276 (45.2%) were group A infections, 334 (54.8%) were group B infections and 21 (3.4%) were A/B co-infections. RSV detection rate is significantly higher (P < 0.0001) in children below 5 years. We noted that the annual distribution of RSV varied substantially by season and for the predominant subtype. Globally, results show a clear circulation pattern in the second half of each year; between June and September and possibly extended into November. The majority of RSV-A strains from Senegal clustered with strains that were previously assigned NA1 and novel ON1 genotype sequences. RSV-B sequences from Senegal clustered with the BA9 genotype. At the amino acid level, RSV-A strains from Senegal show proximity with the genotype ON1 characterized by a 72 nt insertion in G, resulting in 24 extra amino acids of which 23 are duplications of aa 261–283.

**Conclusion:**

Globally our results show a clear circulation pattern of RSV in the second half of each year, between June and September and possibly extending into November, with children under 5 being more susceptible. Molecular studies identified the novel strains ON1 and BA9 as the major genotypes circulating in Senegal between 2012 and 2015.

## Background

Human respiratory syncytial virus (HRSV) is one of the most common pathogens causing severe acute lower respiratory tract infections (ARTIs), accounting for 15 to 40% of pneumonia/bronchiolitis cases in children [1]. It is a non-segmented negative-sense single-strand RNA enveloped virus belonging to the genus *Pneumovirus*. RSV strains are separated into two major groups (A and B) on the basis of antigenic and genetic variability; in each group different genotypes are reported. The main differences are found in the attachment glycoprotein G which interacts with host cell receptors [2]. Eleven RSV-A and 20 RSV-B genotypes have been reported from different geographical regions: (ON1, GA1 to GA7, SAA1, NA1, and NA2) and (GB1 to GB4, BA1 to BA10, SAB1 to SAB4, URU1, and URU2) [3,4] respectively.

Infection with RSV is very common and occurs early in life, usually within the first two years [5]. The virus infects the ciliated airway epithelial cells [6]. The infection can be particularly severe in infants, immunocompromised patients, and elderly individuals [7].

Although the primary role of HRSV in causing infant hospitalizations worldwide is well recognized, the total burden of RSV infection remains poorly defined, especially in African countries.

To address this, we carried out a descriptive and retrospective pilot study, based on the processing of nasopharyngeal aspirates, with a focus on the epidemiology of the HRSV in Senegal after 4 years of surveillance. We also evaluated the genetic variability in the G protein of RSV viruses isolated from clinical samples. Phylogenetic analysis was performed to establish the relationships between Senegalese strains and previously described RSV genotypes deposited in the GenBank database.

## Materials and Methods

### Sample and data collection

From January 2012 to October 2015 nasal-pharyngeal and oral-pharyngeal swabs were collected from consenting Influenza-Like Illness (ILI) outpatients attending sentinel sites (health centers and hospitals). The specimens were collected within 1–7 days of illness onset. Swabs collected were immediately placed at 4°C in 2-mL cryovials containing 1.5 ml cold viral transport medium (Universal Transport Medium; COPAN Diagnostics Inc., Murrieta, CA), and sent to the laboratory. The ILI inclusion criteria were defined according to the CDC case definition as a sudden onset of fever (≥ 38°C) with cough or sore throat lasting fewer than 3 days. For each sample, a case report form on which basic epidemiologic information and symptoms prior to presentation are reported exists. Upon receipt, the specimens were processed immediately for virus detection, identification, and characterization. Aliquots of samples were also stored at −80°C for additional analysis.

Case reports were entered into an Epi Info database (Centers for Disease Control and Prevention, Atlanta, GA) and merged with laboratory data, and frequencies were analyzed using Epi Info.

### Ethical considerations

This study is a component of the 4S network syndromic surveillance [8]. The principles of the 4S network were approved by the Ministry of Health in its guidelines for influenza surveillance policy, finalized with the support of Pasteur Institute in Dakar and the Strengthening Influenza Sentinel Surveillance in Africa (SISA) project funded by the WHO. The protocol and oral consent were determined as routine surveillance activity by the Senegalese National Ethics committee and the steering committee for 4S network, an entity representing MoH, IPD, WHO and Clinicians in compliance with all applicable National regulations governing the protection of human subjects. Data were collected anonymously in an objective of surveillance and applicable to a molecular epidemiology studies on the detected pathogen. The information provided to participants was an informal description of the study. Respiratory specimens were collected, only after informed consent was granted, verbally, to local health care workers by the patients or parents in the case of minors. Oral consent was documented in the patient form with two questions about received information and about oral consent. Patients could refuse to participate, no specimen will be taken. For the surveillance activities, written consent is judged not necessary by the Senegalese national ethics committee, which has also previously approved the work of the National Influenza Center. Collections of non-sensitive data or an observation from normal care in which participants remain anonymous do not require ethics committee review. The patients included in this study were of all ages and consulted the sentinel sites due to influenza-like symptoms; the patients, or parents in the case of minors, accept the tests for respiratory viruses largely because they are free and safe.

### RNA extraction and Detection of respiratory viruses

Total Ribonucleic acid (RNA) was extracted with the QIAmp extraction kit (QIAGEN, Valencia, CA, USA) from a starting clinical specimen volume of 140 μl and final elution volume of 60 μl. RNA was stored at −80°C until use.

For viral detection, RNA was reverse transcribed into cDNA and tested using a multiplex PCR as previously described [8]. The RV16 kit enables direct subtyping of RSV-A and B.

## RSV Molecular Studies

RNA extraction and cDNA synthesis were performed as previously described [8]. For the molecular characterization of HRSV, the second hypervariable region of the Glycoprotein (G) gene was targeted by polymerase chain reaction. The amplification of the gene G protein sequence was carried out by an external and semi-Nested PCR approach.

The external PCR, targeting a 645 bp fragment, was performed with the forward primer ABG490 (5’-ATGATTWYCAYTTTGAAAGTGTTC-3’) and the reverse primer F164 (5’-GTTATGACACTGGTATACCAACC -3’) described in a previous study [9]. Typically the PCR was conducted in a 25μl total volume containing 12.5μl of LongAmp Taq 2X Master Mix (Long Amp Tag 2X Master Mix kit, New England Biolabs), 1μl of each primer (diluted to 10μM), 5 μl of cDNA and 5.5 μl RNase free H_2_O. After a brief initial denaturation of 30s at 94◦C, the PCR was performed using the following conditions: 30 cycles of 94°C for 30 s, 50°C for 30s and 65°C for 1min, followed by a final extension step at 65°C for 10 min and a 4°C hold.

For semi-nested PCR, subgroup A specific primer AG655 (5’-GATCYCAAACCTCAAACCAC -3’) and subgroup B specific primer BG517 (5’-TTYGTTCCCTGAGTATATGTG -3’) were used as forward primers and F164 as reverse primer. One microliter of external PCR was added to a final reaction volume of 25μl. Targeted nested amplicons were 450 bp and 585 bp for RSV-A and RSV-B subgroups, respectively. The nested amplification conditions were as follows: 94°C for 3 min, 40 cycles of 94°C for 30 s, 54°C for 30 s and 68°C for 1 min, with a final extension at 68°C for 10 min and a 4°C hold.

The PCR products were analysed by electrophoresis on a 1.5% agarose gel along with appropriated molecular weight markers (100 bp ladder; New England Biolabs). The gels were then stained with ethidium bromide (0.5μg/ml) before visualization under UV light. In positive samples in either the external or semi-nested PCR, amplicons were cut and purified using the GeneJET Gel Extraction Kit (Thermo Scientific). Purified products were then sent for bidirectional sequencing to Beckman Coulter Services using the same PCR primers. Data in FASTA format were then sent to the laboratory for analysis.

Sequences successfully obtained were aligned with representative GenBank sequences of previously published genotypes using the BioEdit Sequence alignment Editor [10]. The search for sequence similarities was carried out using the Basic Local Alignment Search Tool (Blastn) from the NCBI BLAST web portal. Phylogenetic analyses for both genes (G and F) using the neighbor-joining method, and the statistical significance of the tree topology tested by bootstrapping (1,000 replicates) were performed in MEGA 6 software [11]. The evolutionary distances were derived using the Tamura-Nei method. Bootstrap replicates with values ≥70 are shown on the trees.

The ExPASy (Expert Protein Analysis System) proteomics server of the Swiss Institute of Bioinformatics (SIB) was used for RNA sequence translation. Potential N-glycosylation (Asn-Xaa-Ser/Thr) and O-glycosylation sites were predicted using NetNGlyc 1.0 [12] and NetOGlyc 3.1 [13].

Rainfall and temperature data were collected from the National Meteorological Department of Senegal with the aim to analyze the behavior of the RSV circulation profile with respect to these parameters.

### Statistical analysis

The Chi2 test was used to verify whether gender and patient age criteria, and HRSV infection rates were statistically supported. Tests were made separately for type A, type B and for the 2 types combined. A p value < 0.05 was considered statistically significant. For the age criterion, the [0–5] year age group was used as a reference group. The R.3.0.1 tool was used to perform the analyses.

## Results

### Demographic and clinical details of the enrolled patients

Between January 2012 and June 2015, we enrolled 5338 patients meeting the ILI case definition: 1212 (22.7%) from 2012, 1520 (28.5%) from 2013, 1930 (36.1%) from 2014, and 676 (12.7%) were from 2015. 2634 samples (49.3%) were collected from males and 2672 samples (50%) from females, with a male to female ratio of 0.99. For 32 patients the gender was not reported. Patients’ ages ranged from 1 month to 95 years with a mean age of 10 years 5 months and a median age of 4 years.

In total, 2803 samples (52.5%) were obtained from children younger than five years of age (1294 from girls and 1509 from boys) (Table 1); 1911 samples (35.8%) were obtained from children and adults between 5 and 50 years of age (1009 males and 902 females); 142 samples (2.7%) were obtained from adults above 50 years old. For 482 (9%) patients, the age was not reported.

**Table 1:** Demographical characteristics and symptoms.

Characteristics	Years	2012	2013	2014	2015	Total
		(N = 1212)	(N = 1520)	(N = 1930)	(N = 676)	(N = 5338)
	**Gender** no. (%)					
	**Male**	609 (50.3)	745 (49.0)	936 (48.5)	344 (50.9)	**2634 (49.3)**
	Female	587 (48.4)	767 (50.5)	987 (51.1)	331 (49.0)	**2672 (50.1)**
	Missing	16 (1.3)	8 (0.5)	7 (0.4)	1 (0.1)	**32 (0.6)**
	**Age group** no. (%)					
	0–5 yrs	748 (61.7)	759 (49.9)	941 (48.8)	355 (52.5)	**2803 (52.5)**
	5–10 yrs	117 (9.7)	163 (10.7)	208 (10.8)	83 (12.3)	**571 (10.7)**
	10–15 yrs	68 (5.6)	84 (5.5)	121 (6.3)	41 (6.1)	**314 (5.9)**
	15–25 yrs	71 (5.9)	122 (8.0)	230 (11.9)	61 (9.0)	**484 (9.1)**
	25–50 yrs	59 (4.9)	120 (7.9)	265 (13.7)	98 (14.5)	**542 (10.2)**
	50+ yrs	19 (1.6)	18 (1.2)	83 (4.3)	22 (3.3)	**142 (2.7)**
	Missing	130 (10.7)	254 (16.7)	82 (4.2)	16 (2.4)	**482 (9.0)**
	**Clinical signs** no. (%)					
	Myalgia	124 (10.2)	342 (22.5)	305 (15.8)	95 (14.1)	**850 (15.9)**
	Fever	1197 (98.8)	1463 (96.2)	1927 (99.8)	670 (99.1)	**5257 (98.5)**
	Cough	1024 (84.5)	1299 (85.5)	1654 (85.7)	577 (85.3)	**4554 (85.3)**
	Vomiting	123 (10.1)	33 (2.2)	86 (4.5)	63 (9.3)	**305 (5.7)**
	Diarrhea	93 (7.7)	30 (2.0)	44 (2.3)	12 (1.8)	**179 (3.4)**
	headache	124 (10.2)	183 (12.0)	264 (13.7)	95 (14.1)	**850 (15.9)**
	Dyspnea	20 (1.7)	24 (1.6)	88 (4.6)	12 (1.8)	**144 (2.7)**
	Rhinitis	735 (60.6)	959 (63.1)	941 (48.8)	284 (42.0)	**2919 (54.7)**
	Pharyngitis	609 (50.2)	751 (49.4)	1009 (52.3)	307 (45.4)	**2676 (50.1)**

The most common clinical symptoms were fever (98.5%; 5257/5338), cough (85.3%; 4554/5338), rhinitis (54.7%; 2919/5338) and pharyngitis (50.1%; 2676/5338). No-respiratory symptoms such as vomiting, diarrhea or abdominal pains were noted in some patients.

### RSV detection in the enrolled patients

Of the 5338 samples collected and tested, 610 (11.4%) were positive for RSV as assessed by the real-time RT-PCR method (Table 2): 159 (26.1%) from 2012, 269 (44.1%) from 2013, 164 (26.9%) from 2014 and 18 (2.9%) from 2015. Among the RSV positives, 276 (45.2%) were group A infections, 334 (54.8%) were group B infections and 21 (3.4%) were A/B co-infections. In 34 RSV-positive patients the age was not reported on the case form. During 2012 and 2014 the RSV B type was the most commonly detected (148/159 and 127/164 respectively) while in 2013 type A was most common (224/269).

**Table 2:** Detection rates of human RSV infection in patients with ILI per sub-group and year from 2012 to 2015 in Senegal and comparison of the distribution into the different age groups.

Characteristics	Years	2012	2013	2014	2015	Total
	Total Number	(N = 1212)	(N = 1520)	(N = 1930)	(N = 676)	(N = 5338)
	**Gender** no. (%)					
	Male	609 (50.3)	745 (49.0)	936 (48.5)	344(50.9)	**2634(50.1)**
	Female	587(48.4)	767 (50.5)	987 (51.1)	331(49.0)	**2672(49.3)**
	Missing	16 (1.3)	8 (0.5)	7 (0.4)	1(0.1)	**32(0.6)**
	**Age** no. (%)					
	0–5 yrs	748(61.7)	759(49.9)	941(48.8)	355(52.5)	**2803(52.5)**
	5–10 yrs	117(09.7)	163(10.7)	208(10.8)	83(12.3)	**571(10.7)**
	10–15 yrs	68(5.6)	84(5.5)	121(6.3)	41(6.1)	**314(5.9)**
	15–25 yrs	71(5.9)	122(8.0)	230(11.9)	61(9.0)	**484(9.1)**
	25–50 yrs	59(4.9)	120(7.9)	265(13.7)	98(14.5)	**542(10.2)**
	50+ yrs	19(1.6)	18(1.2)	83(4.3)	22(3.3)	**142(2.7)**
	Missing	130(10.7)	254(16.7)	82(4.2)	16(2.4)	**482(9.0)**
	**Clinical signs** no. (%)					
	Myalgia	124(10.2)	342 (22.5)	305(15.8)	95(14.1)	**850(15.9)**
	Fever	1197 (98.8)	1463 (96.2)	1927 (99.8)	670 (99.1)	**5257(98.5)**
	Cough	1024(84.5)	1299(85.5)	1654(85.7)	577(85.3)	**4554(85.3)**
	Vomiting	123(10.1)	33(2.2)	86(4.5)	63(9.3)	**305(5.7)**
	Diarrhea	93(7.7)	30(2.0)	44 (2.3)	12(1.8)	**179(3.4)**
	Headache	124(10.2)	183 (12.0)	264 (13.7)	95(14.1)	**850(15.9)**
	Dyspnea	20(1.7)	24(1.6)	88(4.6)	12(1.8)	**144(2.7)**
	Rhinitis	735(60.6)	959(63.1)	941(48.8)	284(42.0)	**2919(54.7)**
	Pharyngitis	609(50.2)	751(49.4)	1009(52.3)	307(45.4)	**2676(50.1)**

The RSV detection rate is significantly predominant (P < 0.0001) in children below 5 years of age (436/2803; 15.5%). In the other groups, rates were similar: 6.9% in the 5–10 and 15–25 year old age groups, 3.9% in the 10–15 year old age group and 4.7% in the 25–50 year old age group. Detection in patients above 50 years old was about 0.7%. Gender did not seem important in RSV infections distribution.

We also looked at the distribution of the RSV positive cases inside children under 5 years old (Table 3). We noticed a higher detection rate in the [0,6 months] age group (19.9%; 64/321), similar rates in [6,12 months] and [12,24 months] age groups (16.1% and 16.2% respectively), and lower rate among children aged over 2 years (14%; 184/1318). However these differences are not statistically significant.

**Table 3:** Comparison of the distribution of VRS-positive cases into children under 5 years old age groups.

Characteristics					
	**Age groups**	[0,6]	[6,12]	[12,24]	[24,60]
	**N**	321	484	680	1318
	**VRS/n(%)**	64 (19.9%)	78 (16.1%)	110 (16.2%)	184 (14%)
	**P value**	**RG**	0.852	0.982	0.169

**RG**: Reference Group

Taking into account the clinical symptoms and RSV infection, we noted that cough, rhinitis, pharyngitis and myalgia associated significantly (P < 0.0001) with viral infection.

In order to appreciate the temporal distribution pattern of the HRSV throughout the study period (2012–2015), Fig 1 presents viral detection data on a monthly basis for both viral subtypes (RSV A and B). Rainfall and temperatures (minima and maxima for each month) are also represented. We noted that the annual distribution of RSV varied substantially by season and for the predominant subtype. Globally, results show a clear circulation pattern in the second half of each year; between June and September and possibly extended into November. These periods of increased RSV activity were correlated to rainfall periods and higher temperatures. Year 2013 was an exception, with two RSV waves registered: the first, apparently unusual, occurring early in February and the second in the expected period. As can be seen, each year was characterized by the presence of a dominant RSV type. The other subtype co-circulates at a low level in the population with the possibility of being predominant the next year.

**Fig 1:**
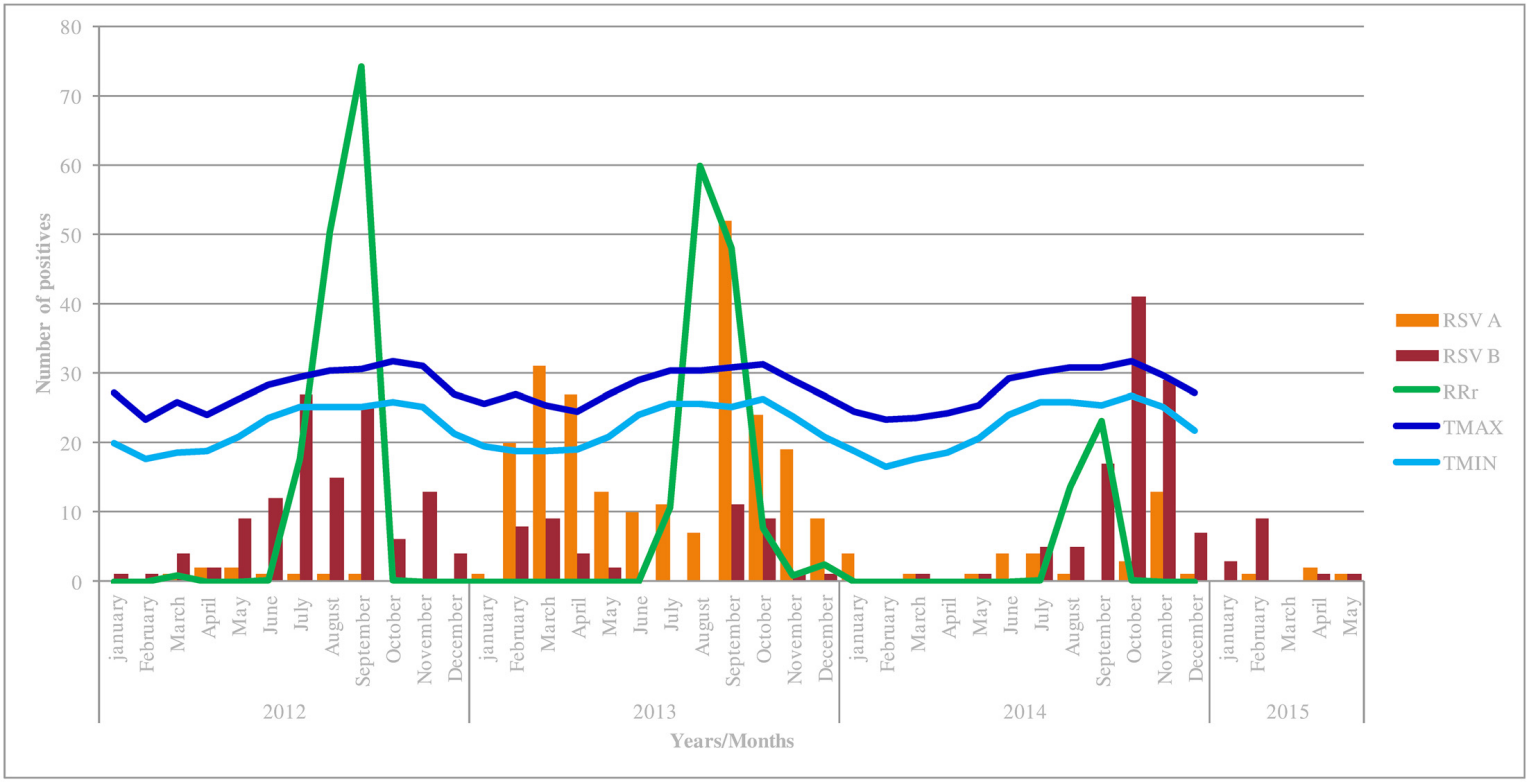
Monthly distribution of RSV A and RSV B infections in patients with ILI in Senegal, over the years of the study (2012–2015). In green is represented the rainfall during the period of study. Temperatures (mean values of the maxima and the minima ones per month) are also represented.

### Phylogenetic comparison of Strains Identified in 2012–2015 in Senegal

For phylogenetic analyses, we were able to obtain partial sequences of the RSV Glycoprotein (G) gene from 64 RSV-positive samples (33 RSV-A and 31 RSV-B). Unfortunately, many samples showed no amplification or poor-quality sequences. The low sensitivity of conventional PCR compared with real time PCR on samples with low viral load, and certainly non-specific amplifications could be the cause of these failures.

**Fig 2:**
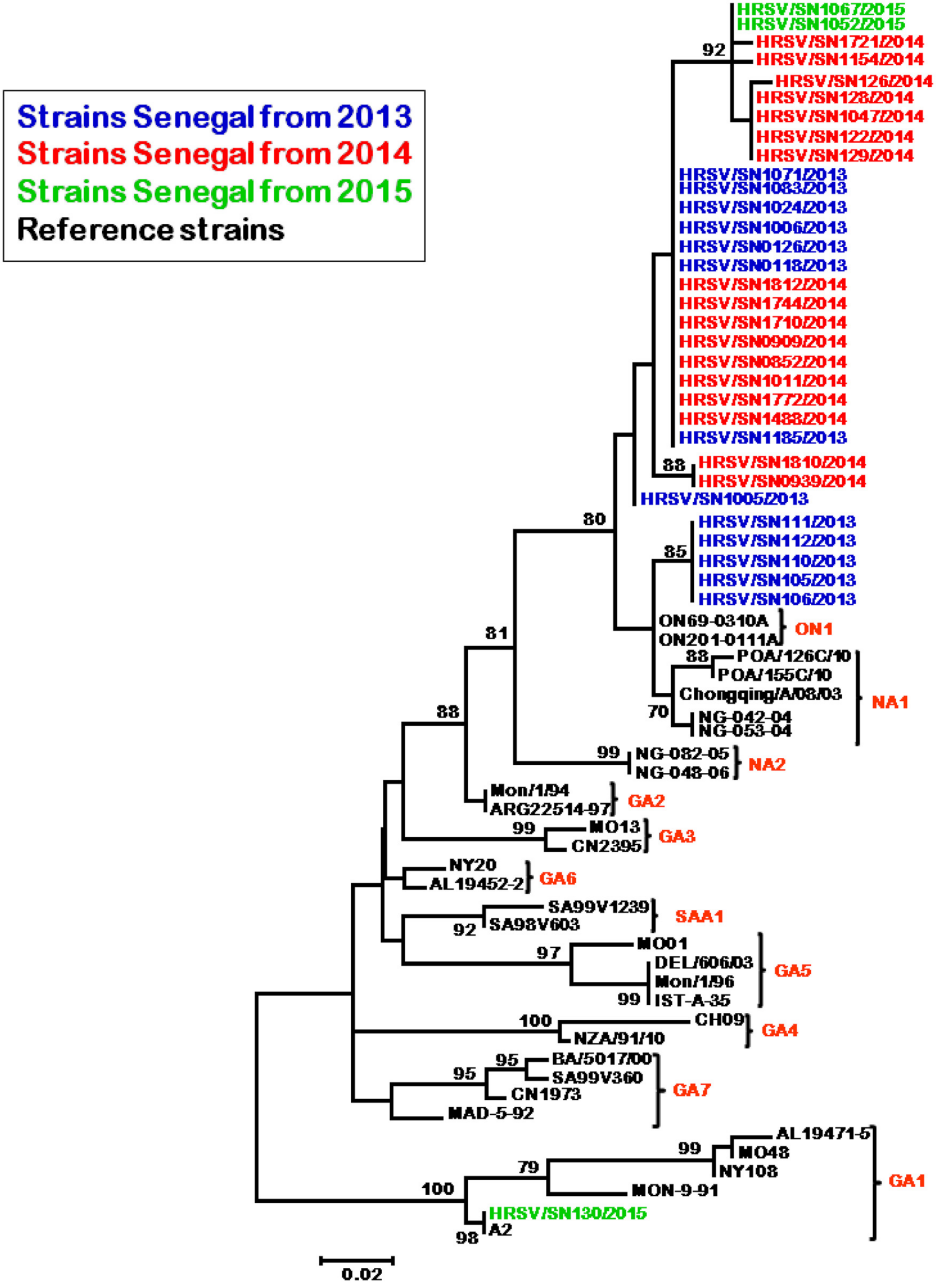
Phylogenetic tree for RSV-A nucleotide sequences strains between 2012 and 2015 based on the second variable region of the G protein. We used the neighbor-joining method with 1000 bootstrap replicates with MEGA 6 version. Senegal isolates are highlighted in different colors for each year and reference strains from Genbank are in black. Only bootstrap values over 70 are shown.

Of the 33 RSV-A sequences obtained, 3 were from samples in 2015, 17 from 2014, and 13 from 2013. The majority of strains from Senegal belonging to subgroup A (32/33, 97%) clustered with strains that were previously assigned NA1 and novel ON1 genotype sequences. This phylogenetic branch, comprising sequences from Senegal samples and sequences from NA1 and ON1 isolates, with a bootstrap value of 72%, clearly stands out from other genotypes (Fig 2). The remaining strain, HRSV/SEN130/2015, seem to belong to the GA1 group with a great proximity to the A2 reference isolate.

**Fig 3:**
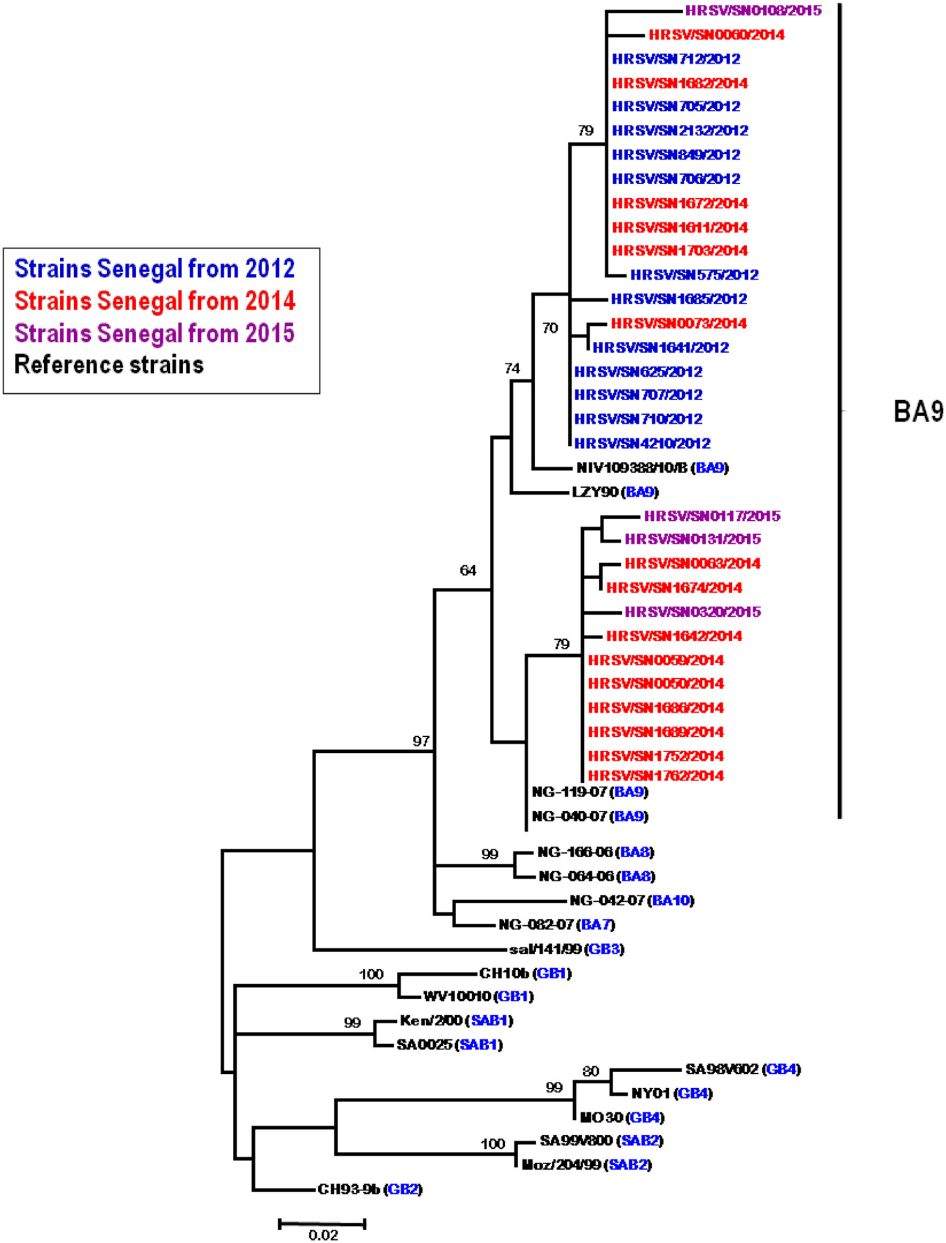
Phylogenetic tree for RSV-B nucleotide sequences strains between 2012 and 2015 based on the second variable region of the G protein. We used the neighbor-joining method with 1000 bootstrap replicates with MEGA 6 version. Senegal isolates are highlighted in different colors for each year and reference strains from Genbank are in black. Only bootstrap values over 70 are shown.

All RSV-B sequences from Senegal (4 from samples in 2015, 15 from 2014 and 12 from 2012) clustered in BA subtype (BA7, BA8, BA9 and BA10) sequences as shown by a phylogenetic branch with a high bootstrap value (Fig 3): 97%. However, it appears clearly that Senegal strains seem to be closest to the BA9 genotype.

### Deduced amino acid sequence analysis of RSV Strains from Senegal

**Fig 4:**
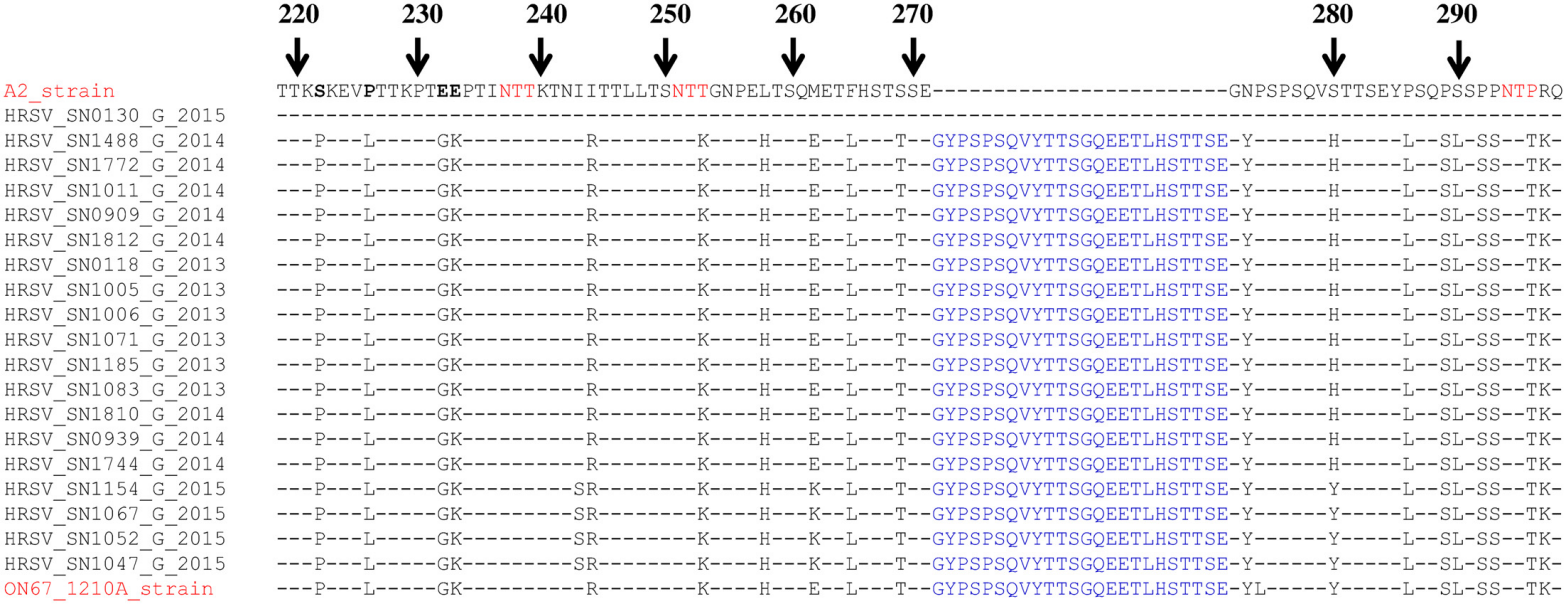
Deduced amino acid alignments of the second variable region of the G protein gene from RSV-A strains from Senegal (in black). Alignments are shown relative to the sequences of prototype strain A2 (GenBank accession number M11486). The amino acid numbering corresponds to strain A2 G protein positions 220 to 308 for the HRSV-A viruses. Identical residues are indicated by dashes. Potential N-glycosylation sites (NXT) in the reference sequence are indicated in red amino acid. Amino acid in blue indicated a duplicated block in strains from Senegal (we voluntary included an isolate from Ontario with the same inserted amino acid block).

We aligned and compared the second hypervariable G region of RSV-A strains from Senegal with A2 and ON67-1210A reference strains (M11486 and JN257693, respectively) (Fig 4).

The major finding was that, at the amino acid (aa) level, RSV-A strains from Senegal show a great proximity with the genotype ON1 (ON67-1210A). ON67-1210A is characterized by a 72 nt insertion in G, resulting in 24 extra amino acids (QEETLHSTTSEGYLSPSQVYTTS) of which 23 are duplications of aa 261–283. The three previously predicted N-glycosylation sites for NA1 strains at aa positions 237, 251 and 294 were also conserved.

Homology between Senegalese RSV-A strains and the novel RSV-A ON1 genotype was about 98% (with 2 substitutions difference) and 97% (with 3 substitutions difference). The unique substitution reported in the ON1 genotype and not the Senegalese strain is the L274P if the amino acid numbering does not include the duplication. Conversely, we noted a unique substitution (I243S) which appears to be specific to 4 strains from Senegal. The strain HRSV/SN130/2015, the unique RSV-A from Senegal without the duplication, showed 100% homology with the reference strain A2.

**Fig 5:**
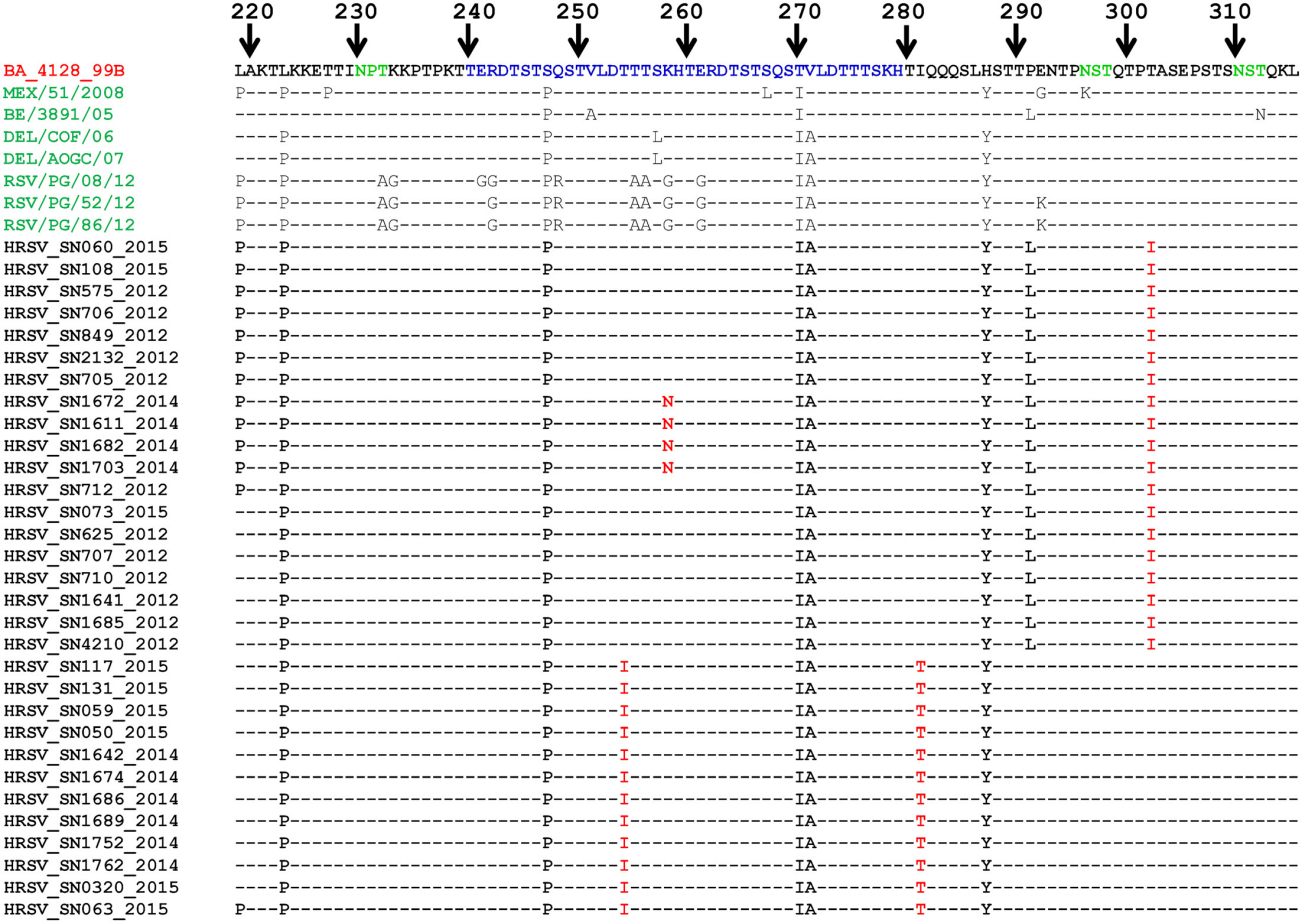
Deduced amino acid alignments of the second variable region of the G protein gene from RSV-B strains from Senegal (in black). Alignments are shown relative to BA4128/99B strain (GenBank accession number AY333364) sequence G protein. Identical residues are indicated by dashes. Potential N-glycosylation sites (NXT) in the reference sequence are indicated in green amino acid. In blue two copies of the duplicated 20-amino-acid. We voluntary introduced other reference strains (in green) and substitutions specific to RSV-B from Senegal are highlighted in red.

The amino sequences of the variable G protein of Senegalese BA strains were also aligned and compared to that of the BA prototype BA4128/99B and other reference strains (Fig 5). They all carry the characteristic 20 aa duplication (TERDTSTSQSTVLDTTTSKH). Furthermore, the alignment showed that S247P and T270I amino acid substitutions observed in the duplicated 20 amino acid region were found in all Senegalese BA strains. BA strains from Senegal share many substitutions with other reference strains: L219P, L223P, V271A, H287Y and P291L. Besides that, some aa substitutions (in red) appear specific to Senegal BA strains. Three potential N-linked glycosylation sites were also identified in all our strains: the first site is located at aa 230, a second site is located at aa 296 and a third one at aa 310 position.

## Discussion

The current study is the first to have investigated the molecular epidemiology of RSV in Senegal and even in West Africa. The study was based on a 4 year period of surveillance of outpatients, with a total of 5338 specimens from ILI patients of diverse ages having been collected and laboratory tested for RSV infection.

The overall RSV detection rate is 11.4%. This proportion of RSV detection among patients with ILI is consistent with that of studies conducted in other countries, including Gabon (11.9%) [14], Kenya (9.1%) [15], Philippines (11.1%) [16] and Colombia (8.9%) [17]. The lowest detection rates have been registered in the USA (7.7%) [18], North Korea (2.7%) [19], Brasil (2.4%) [20] and Cameroon (5.7%) [21]. However, a study conducted in Madagascar [22] on 313 patients with ILI revealed the highest RSV attack rate of 21.2%. These discrepancies in RSV detection rates among patients with ILI, beyond technical approaches, can be attributed to geographical differences in virus burden, the number of patients tested, the periods during which samples were collected and even the duration of the study.

In our study, the majority (71.5%; 436/610) of RSV-positive patients were children between 0 to 5 years old. These results are in line with observations reported in previous studies. Indeed, RSV represents a substantial burden of acute respiratory tract illness particularly in the early years of life, leading to severe morbidity and hospitalization in very young children [23,24,25]. In relation to the sex of patients, even if previous studies suggest that male children are more susceptible to severe disease than females [5,9,26], statistical analysis on clinical features and detection rates between male and female patients did not reveal any striking differences in our study.

Our surveillance data confirmed that the RSV season peaked between July and November each year, with variations on the exact beginning month and the duration of peaks in different years. However, ongoing low-level circulation outside of the epidemic season was detected, which probably contributes to the persistence of strains between seasons. We also found that the incidence of RSV correlated positively with the rainfall peak and temperature increase. RSV is an enveloped virus, and higher relative humidity following the rainfall appears to be an advantage for its persistence in the environment. Indeed, it has been well demonstrated [27] that the survival of the RSV in tropical regions is highest in high relative humidity and high stable temperatures, allowing large droplet aerosols to sustain the transmission of the virus in these populations throughout the year. However in other areas, it was showed that RSV incidence was highest during the cooler months [28,29].

We evaluated RSV genetic variability in nasopharyngeal swab samples taken from patients with ILI from Senegal during 2012–2015. Phylogenetic analysis revealed that the majority of RSV-A strains sequenced (32/33) clustered with strains of the novel ON1 genotype first described in Ontario, Canada in 2010 [4]. The novel ON1 genotype is characterized by a 72 nucleotide duplication in the C terminal third of the G gene which induced codon disruption and lengthening of the subsequent predicted polypeptide by 24 AAs, including 23 duplicated AAs. Based on our results, this genotype would be present in Senegal before 2013. In line with other findings from Europe, other African countries and Asia, this study reports ON1 as the predominant genotype during the RSV 2012–2015 surveillance, suggesting a rapid spread of this emerging RSV strain around the world [30].

Sequence and phylogenetic analysis revealed that all RSV-B subtype strains from Senegal belonged to the genotype BA, which is characterized by 60-nucleotide duplication in the secondary hypervariable region of the G gene [31]. This hypervariable region is characterized as a target for genotype-specific neutralizing antibodies. However, we exclusively found one circulating BA genotype in our study: BA9. This genotype belongs to the new BA group genotypes (BA7, BA8, BA9, and BA10) which were previously classified as BA4 [32].

The study’s limitations include first that it was designed to address influenza–associated burden in patients with ILI in Senegal. A recent study [33] reported that being limited to the ILI case definition (and even SARI) would underestimate the RSV burden in children. The authors supported that the sensitivity and specificity for detection of RSV–associated cases were highest with ARI standard definition cases, and respiratory signs like a wheeze and tachypnea were more likely associated with RSV infection. Secondly, in our study only outdoor patients were recruited. Thus further studies including both outpatients and inpatients (hospitalized) would be required to firmly establish the burden associated with RSV in Senegal. The number of strains sequenced in our study should also be more exhaustive for each year in order to get the full spectrum of RSV strains that may be circulating in ILI patients in Senegal.

## Conclusion

Globally our results show a clear circulation pattern of hRSV in the second half of each year, between June and September and possibly extending into November. These periods of increase in RSV activity were correlated to rainfall periods and an increase of temperatures. Our findings also identified children aged under 5 as being more susceptible to RSV infection. Molecular studies identified the novel strains ON1 and BA9 as the major genotypes circulating in Senegal between 2012 and 2015.

Further genotyping and molecular epidemiological surveillance of RSV with whole genome sequencing of 0N1 and BA9 genotypes detected in Senegal should be carried out. This would enhance understanding of the local and global molecular epidemiology and phylogeography of RSV strains from Senegal. The next step will be the assessment of the burden of hRSV among children in Senegal focusing on pediatric hospitalized cases. Data on disease outcome, atypical clinical signs, duration of symptoms, duration of hospitalization and treatment will be collected for a better burden assessment.
